# Imported pediatric malaria at the hospital for sick children, Toronto, Canada: a 16 year review

**DOI:** 10.1186/1471-2431-14-251

**Published:** 2014-10-04

**Authors:** Andrea B Evans, Dina Kulik, Anna Banerji, Andrea Boggild, Kevin C Kain, Mohamed Abdelhaleem, Shaun K Morris

**Affiliations:** Department of Pediatrics, University of Toronto, Toronto, Canada; Department of Pediatric Emergency Medicine, Hospital for Sick Children, Toronto, Canada; Department of Global and Aboriginal Health, Continuing Education and Professional Development, Faculty of Medicine, University of Toronto, Toronto, Canada; Tropical Disease Unit, University Health Network-Toronto General Hospital, Toronto, Canada; Tropical Disease Unit, SAR Labs, Sandra Rotman Centre for Global Health, University Health Network-Toronto General Hospital, Toronto, Canada; Department of Haemopathology, Hospital for Sick Children, Toronto, Canada; Division of Infectious Diseases, Hospital for Sick Children, 555 University Ave, Toronto, ON M5G1X8 Canada

**Keywords:** Malaria, Pediatric, Visiting friends and relatives, Immigrant health

## Abstract

**Background:**

Children under 5 represent 86% of annual malaria deaths in the world. Following increasing trends in international travel, cases of imported malaria are rising in North America. We describe the epidemiology of malaria diagnosed at a tertiary care pediatric center in the multicultural city of Toronto.

**Method:**

Retrospective chart review of all laboratory confirmed malaria from birth to <18 years between July 1, 1997 and June 30, 2013. Epidemiological data, travel history, chemoprophylaxis history, as well as clinical presentation, diagnosis and treatment were extracted.

**Results:**

In total 107 children were diagnosed with malaria in the 16 year time period. *Plasmodium falciparum* malaria was identified in 76 (71%), *Plasmodium vivax* in 28 (26%). Median age of infected children was 6.7 years where 35% of children were born in Canada, 63% were recent or previous immigrants. Of those who resided in Canada, reason for travel included visiting friends or relatives (VFR) 95% and tourism or education (5%). Most common countries of infection were Ghana (22%), Nigeria (20%) and India (14%). Median parasitemia at presentation to our institution was 0.4% (IQR 0.1-2.3) with a maximum parasitemia of 31%. Nineteen (18%) met the WHO criteria for severe malaria due to hyperparasitemia, with 3 of these cases also meeting clinical criteria for severe malaria. One third of patients had a delay in treatment of 2 or more days. Ten percent of children had seen two or more primary health care professionals prior to admission. Prophylaxis was documented in 22 (21%), and out of those, 6 (27%) were appropriate for the region of travel and only 1 case was documented as adherent to their prescription. There were no cases of fatality.

**Conclusion:**

Malaria continues to be a significant disease in returning travelers and immigrant or refugee populations. An increase in physician awareness is required. Appropriate pre-travel advice, insect protection measures, effective chemoprophylaxis is needed to reduce the incidence and improve the management of imported pediatric malaria.

## Background

Despite being largely preventable and treatable, malaria is estimated to kill 660 000 to 1 240 000 people per year with children under 5 representing 86% of annual malaria deaths in 2010 [1–3]. Although there is no longer endemic transmission in Canada, malaria may be acquired abroad by travelers to endemic regions. Imported malaria is defined as malaria infection acquired in a malaria endemic area but diagnosed in a non-endemic country [4, 5]. Globally, there has been a dramatic rise in the number of imported malaria cases in non-endemic countries, particularly caused by *Plasmodium falciparum* [2, 3, 6, 7]. This increase in diagnosed imported malaria follows closely trends in increasing international travel and immigration [6, 7]. The majority of new malaria cases in North America now stem from travel to endemic countries to visit friends and relatives (VFR) [8–11].

Children account for 15-20% of imported malaria cases and present distinctly from adults with malaria [8, 12]. Importantly, children have different clinical presentations, are at higher risk of developing severe disease, and have an increased likelihood of death compared to adults.

Despite the growing threat of imported malaria, there have been few studies in North America, evaluating the epidemiology of imported malaria in children [7, 13]. This represents a significant gap in the understanding of imported malaria in children. Toronto is one of the most diverse city in the world; in the 2006 census, 45.7% of the city’s population was foreign-born [14] and in 2011, and had 1.36 million overseas visitors [15]. The Hospital for Sick Children in Toronto (SickKids) is a tertiary care centre that is Canada’s largest children’s hospital and is located in downtown Toronto. According to the Canadian Institute for Health Information (CIHI) database, between 2002–2012 there were 242 cases of pediatric malaria in the province of Ontario (61% of all Canadian cases), of which 23% were seen at our institution [16].

The objectives of the current study are twofold. First, describe the epidemiology of malaria diagnosed at SickKids over a 16 year period from 1997 to 2013. Second, identify populations at increased risk for malaria infection and at risk of severe malaria in Toronto. Understanding the epidemiology of imported malaria will support the design and implementation of targeted interventions for young travelers to endemic countries and others at risk both in Toronto and in similar large multi-cultural cities.

## Methods

This study is a retrospective review of all cases of laboratory-confirmed malaria from birth to <18 years, at SickKids between July 1, 1997 and June 30, 2013. Ethics was approved by the SickKids Institutional Review Board and access to clinical records were granted by Medical Records at SickKids, and laboratory records were granted by the Department of Microbiology. A positive malaria test result was defined as positive thin or thick smear or a positive rapid malaria test (BinaxNOW Malaria Test, Binax Inc., Scarborough, ON), BinaxNOW histidine-rich protein 2 test (T1) and BinaxNOW aldolase (T2) test were both conducted. Cases were identified through two methods: 1) SickKids microbiology records of positive thin or thick smears, and 2) ICD-9/10 codes from SickKids medical records. All positive thin or thick smears of malaria are sent to the Public Health Ontario Laboratories for confirmation. We included as cases only those which the Public Health Ontario Laboratory confirmed as positive by smear. There was a single case where the diagnosis of malaria was given to a patient (negative smears) due to positive rapid malaria test done at SickKids. This case had negative smears and negative rapid malaria test at PHL, and was not included in our study.

After identifying cases, all medical charts were reviewed for demographic, history, laboratory, treatment and intervention data.

Migration history extracted from the chart included 1) traveler/tourist from or to Canada, 2) a resident of Canada who was travelling abroad visiting friends or relatives 3) a recent immigrant to Canada 4) a recent refugee to Canada.

A person whose travel originated from Canada, and whose purpose or the type of accommodation involved visiting friends and/or relatives, as documented in the chart was defined as visiting friends or relatives (VFR). An immigrant was defined as a person who was born outside of Canada, who has arrived to Canada for permanent residency and was not a refugee claimant. An immigrant on recent arrival was defined as an immigrant arriving to Canada at the visit considered in this study as being the point of exposure to malaria. An immigrant at a previous arrival was defined as a patient who arrived in Canada prior to the visit considered in this study as being the point of exposure to malaria. A refugee was defined as a person who had refugee status as documented by Interim Federal Health or on history taken by a healthcare worker.

We defined ‘appropriate’ chemoprophylaxis as documentation that a patient was prescribed a anti-malarial prophylaxis regimen that was appropriate for planned travel itinerary. Severe malaria in this manuscript is defined based on WHO definition of severe malaria [17] which includes the criteria of hyperparasitemia. Hyperparasitemia is based upon the WHO definition of parasitemia >2%, or >5% in semi-immune population. Median delay in treatment was defined as days between presentation to any physician and treatment. Non-immune population was defined as a patient born in Canada and traveling to an endemic country (as tourist or VFR). A semi-immune population for the purposes of this paper was defined as an immigrant on recent arrival.

Data was analyzed using SPSS (2012 v.21, Armonk, NY: IBM Corp). Statistical analysis of data was conducted using Kolmogorov-Smirnov Test for normality of distribution and Levene’s test was used to determine equality of variance. For variables with normal distribution student’s *t*-test was used to compare differences between two independent groups. Chi-squared test was used for categorical variables and Mann–Whitney U was conducted for continuous variables to compare differences between two independent groups where distribution was significantly different from normal. Results were considered significant when the probability of making a Type I error was <5% (p <0.05).

## Results

One-hundred and seven children were diagnosed with malaria in our institution between July 1, 1997 and June 30, 2013. There was a median of 6.5 (interquartile range (IQR) 4–10) cases per year with no appreciable trend over the 16 years. *P. falciparum* was identified in 76 (71%), *P. vivax* in 28 (26%), *Plasmodium oval*e in 2 (2%), and *Plasmodium malariae* in 1 (1%). The median age of infected children was 6.7 years (IQR 2.8 – 11.7) and 69 (64%) were boys (Table 1). There were 2 pairs of siblings in our study. 19 (18%) children met the WHO criteria for severe malaria. One 1.1 year old child met the WHO criteria for cerebral malaria with *P. falciparum*, and one 4 week old child had congenital malaria with *P. vivax*.

**Table 1:** **Demographics**

Demographics	Number
Total	107
Male	69 (64%)
Age (median)	6.6 (IQR 2.8-11.7)
Status in Canada known	99 (92%)
*Canadian born*	35 (35%)
*Immigrant*	53 (54%)
Immigration date known	33 (62%)
Immigrant (this arrival)	25 (47%)
Immigrant (previous arrival)	8 (15%)
*Refugee Claimant*	7 (7%)
*Adopted*	2 (2%)
*Visiting Canada in transit*	2 (2%)
Reason for Travel from Canada	43 (40%)
(Canadian-born and previous immigrants)	
Visiting Friends or Relatives	41 (95%)
Tourism/Education	2 (5%)
Prophylaxis History Documented	22 (20%)
*Type of prophylaxis*	
Mefloquine	6 (27%)
Chloroquine	5 (23%)
Other	2 (9%)
None	9 (41%)
*Adherence*	
Adherent	1 (5%)
Non-adherent	21 (95%)

IQR is interquartile range.

Out of the 99 (93%) of cases where country of birth was documented, 35 (35%) children were born in Canada. Sixty-two (63%) cases were recent or previous immigrants, 7 (7%) were documented as refugee claimants, 2 (2%) were recently internationally adopted children. Out of the 43 children that were documented to have had travel originating in Canada (Canadian born or previous immigrants), 41 (95%) were visiting friends or relatives (VFR) and 2 (5%) were traveling for tourism or studying abroad. Children with *P. falciparum* infection were more likely to be born in Canada than those infected with *P. vivax* (p = 0.01).

The most common locations of infection were West Africa (Ghana 24 (22%), Nigeria 21 (20%)), and South Asia (India 14 (13%), Pakistan 12 (11%)) (Table 2).

**Table 2:** **Region and country of exposure**

Region	Country	n
Africa	Ghana	24
	Nigeria	21
	Ivory Coast	5
	Democratic Republic of Congo	3
	Cameroon	3
	Tanzania	3
	Kenya	2
	Uganda	2
	Ethiopia	2
	Angola	1
	Benin	1
	Congo	1
	Republic of Guinea	1
	Mali	1
	Rwanda	1
	Zambia	1
South Asia	India	14
	Pakistan	12
	Thailand	2
South East Asia	Sri Lanka	1
	Afghanistan	1
Central America	Honduras	2
	Nicaragua	1
South America	Guyana	3

Out of the 22 cases where there was documentation of the use of chemoprophylaxis, 9 (41%) were not prescribed any prophylaxis. Of those who were prescribed prophylaxis, 5 (23%) took chloroquine, 6 (27%) took mefloquine, one case took sulfadoxine and pyrimethamine, and one case took amodiaquine prior to arrival at the hospital. Appropriate prophylaxis for the region of travel was prescribed in 6 (27%) of cases, all but one of these cases recorded adherence to medication, however only in one case documented adherence to the medication was as directed. Documentation of past malarial history was available for 91 cases, of which 34 (37%) had reported previous episode of malaria.

The most common symptoms described on history were fever (100%), vomiting (32%), headache (22%), and chills (20%). Fever was documented in the emergency room on presentation in 40% of cases, tachycardia in (20%), hepatosplenomegaly in 20%, hypotension in 5%, and CNS impairment (decreased level of consciousness, fainting, or seizures) in 3%. Table 3 compares characteristics of children infected with *P. falciparum* and those infected with *P. vivax*. There was no statistically significant difference in symptoms or signs between the groups. Notably, children infected with *P. falciparum* were more likely to have had more days of symptoms than those infected with *P. vivax* (p = 0.01).

**Table 3:** **Characteristics of cases of** ***P. falciparum*** **and** ***P. vivax*** **malaria**

	***P. falciparum***	***P. vivax***	p value
Number of cases	76 (70%)	28 (26%)	
Age (median)	9.05	5.4	*0.01*
Male	45 (59%)	21 (75%)	*0.047*
Top 3 countries of origin	Ghana (32%)	Pakistan (38%)	
	Nigeria (28%)	India (35%)	
	Ivory Coast (7%)	Honduras (8%) and Thailand (8%)	
Born in Canada	28 (37%)	3 (11%)	*0.01*
Immigrant or Refugee	48 (63%)	23 (82%)	*0.01*
VFR	39 (51%)	9 (32%)	0.25
Tourism/Education	2 (3%)	0 (0%)	*0.01*
Length of trip (weeks) (median)	5 (IQR 9–10)	8 (IQR 3–36)	0.57
Days since arrival in Canada	14 (IQR 7–21)	30 (IQR 16–270)	*0.01*
Days of Symptoms (median)*	4.0 (IQR 2–7)	6.5 (IQR 3–11.5)	*0.01*
Delay to Treatment (days) (mean)**	1.6 (2.4)	3.7 (5.6)	0.27
Symptoms on Presentation to ED			
Headache	14 (18%)	8 (29%)	0.28
Vomiting	24 (32%)	9 (32%)	0.62
Abdominal Pain	10 (13%)	3 (11%)	0.92
Diarrhea	8 (10%)	1 (4%)	0.75
Cough	9 (12%)	2 (7%)	0.90
Anorexia	6 (8%)	3 (11%)	0.85
Malaise	10 (13%)	4 (14%)	0.50
Chills	12 (16%)	8 (29%)	0.16
Signs on Presentation to ED			
Fever	28 (37%)	11 (39%)	0.31
Tachycardia	19 (25%)	2 (7%)	0.30
Hypotension	6 (8%)	1 (4%)	0.51
Hepatosplenomegaly	17 (22%)	4 (14%)	0.50
CNS impairment	4 (5%)	0 (0%)	0.52
Parasitemia (percent) (median)	0.8 (IQR 0.1-5)	0.3 (IQR 0.1-0.9)	*0.03*
WHO defined severe malaria	19 (25%)	0 (0%)	*0.00*
Lowest hemoglobin (median) (mg/dL)	91.5 (IQR 75–104)	111 (IQR 97–126)	*0.01*
Lowest platelet (mean) (10^3^/μL)	116 (116)	105 (57)	0.28
Length of stay in hospital (days)	3.0 (IQR 1–4)	1.0 (IQR 1–3)	*0.02*
Intensive care admission	5	0	*0.01*

*Number of days of symptoms at presentation in Emergency Department (ED) at SickKids.

**Delay to treatment is defined as time from presentation to any physician and treatment.

Median parasitemia was 0.4% (IQR 0.1 – 2.3), where the maximum parasitemia seen was 31%. Forty-six (43%) cases had parasitemia of = <0.1%. Of those with parasitemia >0.1%, the median parasitemia was 1% (IQR 0.4 – 5). There was a greater median parasitemia in children with *P. falciparum* infection compared to *P. vivax* infection (p = 0.03). One quarter (19) of children presenting with *P. falciparum* had severe malaria. All patient satisfying the WHO criteria for severe malaria had hyperparasitemia, and three cases had additional clinical features (decreased level of consciousness, seizures, and circulatory collapse (BP < 70 systolic)). There were no significant differences in time away from Canada, time to presentation, delay in treatment, hospital stay between patients with non-severe and severe malaria.

Binax test results performed by the Ontario Public Health Laboratory were available for 42 patients (23 with *P. falciparum*, and 16 with *P. vivax*) between the years of 2006 to 2013. Binax T1 was positive for 22 cases (21 (95%) of which had *P. falciparum*) and Binax T2 was positive for 29 (16 (55%) of which were infected with *P. falciparum*). Binax T1 tests were negative for 2 children who were confirmed to have *P. falciparum* on blood smear. Similarly, Binax T2 tests were negative for 4 patients who were confirmed to have *P. vivax*. Binax T1 Sensitivity for *P. falciparum* 91.3%. Binax T2 sensitivity for *P. falciparum* 69.6%. Binax T1 is not sensitive for the detection of *P. vivax* whereas Binax T2 has a sensitivity of 75% for *P. vivax*. Our data is limited to patients who had positive microscopy for malaria, thus specificity cannot be calculated.

Children infected with *P. falciparum* were more likely to have a hemoglobin < 120 mg/dL (120 g/L) than those infected with *P. vivax* (p = 0.01). No child had severe anemia as defined by the WHO criteria for severe anemia (hct <15%, hgb < 50 mg/dL). Mean platelet count for those presenting with *P. falciparum* was 116 × 10^3^/μL, and for those presenting with *P. vivax* was 105 × 10^3^/μL there were no statistically significant difference between groups.

Children infected with *P. falciparum* were treated with single agent or combination of quinine (69%), atovaquone-proguanil (32%), sulfadoxine-pyrimethamine (16%), doxycycline (13%), clindamycin (22%), or single agent artesunate (5%). Intravenous quinine was used in the emergency room even for patients eventually discharged and treated subsequently with PO quinine as outpatients. Non-falciparum malaria infections were treated with single agent chloroquine (10%) or combination agents doxycycline and quinine (12%) and primaquine and chloroquine (46%) or primaquine and atovaquone-proguanil (32%). Sulfadoxine-pyrimethamine was predominantly used prior to the year 2001 and atovaquone-proguanil after 2001. Antimalarial treatment was refused in one case of an 8.5 year old boy exposed in Pakistan a year prior to presentation with 0.1% parasitemia of *P. vivax*. This child was followed up over the telephone after not presenting to the follow-up infectious disease clinic. Parents had preferred treatment with tincture of quinine despite a prescription of primaquine. Further telephone follow-up indicated the child was asymptomatic with no adverse events.

Five patients (5%), all *P. falciparum*, were transferred to the pediatric intensive care unit. Of these five patients, length of stay ranged from 3–5 days. In total, nine patients received blood transfusions, 1 patient received exchange transfusion, 1 patient refused blood transfusion due to religious beliefs. There were no reported cases of mortality.

Table 4 summarizes inpatient and outpatient data for all cases. The majority of patients were treated as inpatients with 89 (83%) admissions to the hospital. Median hospital stay was 2 days (0.5 – 57 days, interquartile range 1–4) with 12% of patients staying greater than 4 days in hospital. Children infected with *P. falciparum* had longer stay compared to *P. vivax* (p = 0.02)*.* Of those 9 patients who were treated as outpatients with *P. falciparum*, only one patient had percent parasitemia >0.1%. This patient was an 8 year old girl who had a parasitemia of 2%, arrived from Nigeria 2 days prior to presentation and was claiming refugee status. She was given IV quinine prior to discharge from the emergency department (ED), and follow-up with the Infectious Disease team at SickKids within 2 days.

**Table 4:** **Outpatient and inpatient characteristics of malaria cases**

	Outpatient	Inpatient
Number of cases	18 (14%)	89 (83%)
Age	9.8 [5.3-13.8]	7.0 [2.8-10.8]
Male Gender (%)	67%	63%
Transfer from outside institution (%)	5%	50%
Status in Canada Known	12 (67%)	87 (98%)
*Canadian born*	3 (25%)	*32 (30%)*
*Immigrant/refugee*	*9 (75%)*	44 (48%)
Immigration date known	7 (78%)	26 (29%)
Immigrant/refugee (this arrival)	4 (44%)	21 (24%)
Immigrant (previous arrival)	3 (33%)	5 (6%)
*P. falciparum*	9 (50%)	66 (76%)
Median parasitemia (%)	0.1 [<0.1-2.0]	3.3 [0.28-3.8]
Mean Hemoglobin level	112 (20)	93 (23)
Median days to follow-up appointment in ID clinic	2 [1-7]	10 [7-21]

ID clinic = Infectious Disease outpatient clinic at SickKids.

Data in square brackets represent IQR. Data in curved brackets represent SD.

Blood cultures were routinely collected for patients being admitted to hospital. Broad spectrum antibiotics was administered for 35 (33%) of patients, of which 3 (3%) eventually had a confirmed bacterial infection (two cases of non-typhoidal salmonellae bacteremia, one case of *E. coli* urinary tract infection). There were 10 cases that had a primary diagnosis other than malaria and had low malaria parasite load (<0.1%).

**Figure 1:**
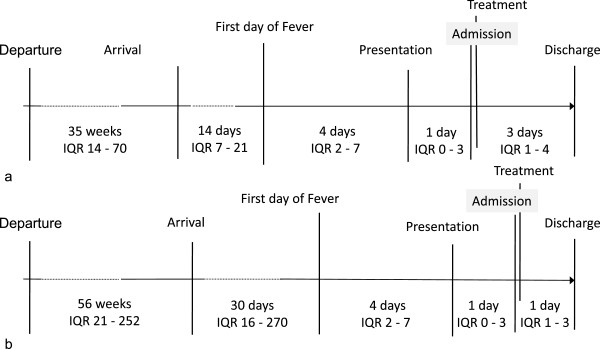
**Timeline of events leading to presentation and treatment of malaria a)** ***P. falciparum*** **b)** ***P. vivax*** **.** Data represents median and interquartile range (IQR). Departure is defined as departure from Canada for subjects leaving Canada to another country. Arrival is defined as the approximate time of arrival in Canada for all subjects. Presentation is defined as the first day of presentation to any physician.

Figure 1 is a timeline illustrating travel history and important events for all patients. Median time in Canada prior to symptoms was 13 days (IQR 10–41), and median time of travel if not a first time immigrant was 42 days (IQR 21–140) and the maximum time of travel was 504 days. Median delay to treatment, was 1 day (IQR 0–3) and the maximum was 23 days. However, one third of patients had a delay to treatment of 2 or more days. Median days between first symptom attributed to malaria and contact with the health system, was 5 days (IQR 2–9). Thirty-eight percent of patients had seen a primary health care professional before admission to SickKids, and 10% had seen two or more primary health care professionals prior to admission.

## Discussion

Imported pediatric malaria remains an important cause of fever in returning travelers. Our study is consistent with other case reviews revealing that travelers visiting friends and relatives contribute the bulk of imported cases. Use of chemoprophylaxis was low and in only one case did we find that the appropriate drug was prescribed for the region of travel and that the patient reported proper adherence. This emphasizes that awareness amongst both travelers and primary care physicians play an important role in malaria prevention by providing pre-travel counseling and prescription of appropriate chemoprophylaxis along with non-pharmaceutical prevention of malaria (behavior modification, insecticide-treated bed nets, and insect repellents). Physicians can refer to up to date chemoprophylaxis information from the Center for Disease Control Yellow Book “Health information for International Travel 2014”: <http://wwwnc.cdc.gov/travel/yellowbook/>and the World Health Organization treatment guidelines: <http://www.who.int/malaria/>, or the published guidelines form the Committee to Advise on Tropical Medicine and Travel (CATMAT) public health agency of Canada <http://www.phac-aspc.gc.ca/publicat/ccdr-rmtc/>.

We also report the nonspecific signs and symptoms of malaria presentation of which fever is most often present in the history. However documentation of a fever in the ED was only present in 40% of our cases. Further, travel history was often remote particularly in those infected with *P. vivax*, where 20% of patients were in Canada more than 2 months prior to presentation. Many patients had seen multiple primary care physicians (12% were seen by two or more physicians prior to arrival at SickKids where thin and thick smears were undertaken and treatment was initiated). Primary care and ED physicians should be aware that vital signs and physical examination are often times nonspecific and that travel history may be remote.

The most common regions of acquisition of malaria reported in our study are consistent with those in the literature. In general, the top source country for newcomers in Canada are China, India, Pakistan. Source countries (between the year 2000 and 2013) with high risk of malaria are in order: Nigeria, Afghanistan, Cameroon, Democratic Republic of Congo, Guyana, Ghana, Rwanda, Uganda, and Honduras. In general, countries with English, such as Guyana, or French as a predominant language such may be choice countries for Canadians to travel or for tourism or education and as such may be overrepresented in our data set even though the risk of acquisition of malaria is by comparison low in these countries [14, 15].

The BinaxNOW results reported in this study are consistent with reported sensitivities and specificities of this test in the literature [18, 19]. This test is thus an alternative for malaria diagnosis where hematopathology is not available. However, it is worth noting that BinaxNOW and rapid diagnostic tests for malaria, in general, are insufficient to exclude non-falciparum malaria, due to poor sensitivity in malaria due to P. vivax, P. malariae, or P. ovale [18, 20, 21]. Our data on BinaxNow is limited by the fact that this test was used only between 2006 and 2013, and we only have data for those subjects who had positive microscopy for malaria. Thus we do not report false positive results. It should be kept in mind as well that examination of thick and thin blood smears is the only available laboratory test which can reliably differentiate clinically relevant asexual parasitemia from clinically irrelevant sexual parasitemia.

According to the ‘Evidence-based clinical guidelines for immigrants and Refugees’ [22], there is no need to conduct routine screening for malaria. The two thirds of patients seen or admitted to SickKids were new immigrants/refugees arriving into the country from endemic regions, therefore unlikely to be on malaria prophylaxis. If these children are not screened for malaria on arrival, there must be a high index of suspicion for malaria with any systemic illness. Furthermore, there is no institutional policy within our hospital to screen siblings. However, when there is a family history of travel and the patient is very ill siblings are generally evaluated to make a decision on performing a thin smear, at the discretion of the ordering physician. As reported by our study, children with malaria often present with non-specific symptoms such as fever, lethargy, malaise, vomiting, abdominal pain and diarrhoea [23]. Hepatomegaly, splenomegaly and jaundice are often present if detected on physical examination, affecting 40-60% of children with malaria [23]. Common hematologic abnormalities include anemia occurs in 30-100% of cases and thrombocytopenia is present in 45-75% of imported malaria cases [24–26]. Those infected with *P. falciparum* usually present within one month of travel, while those with *P. ovale* or *P. vivax* infections can present years later [26, 27].

The other major group in this study were the VFR children who are a group that should be targeted for pre-travel advice. These children VFRs are less likely to seek pre-travel health advice, take anti-malarial prophylaxis or take bite-prevention measures [4, 8, 9, 12, 26, 28]. Studies demonstrate that approximately 60% of VFR take no prophylaxis while 15-20% take inappropriate prophylaxis [8, 29–33]. As this study was a retrospective chart review where prophylaxis history was not a mandatory question when seen by a healthcare professional, it is likely that data has limitations in fully reporting prophylaxis adherence. However, in our documentation, 25% of chemoprophylaxis was properly prescribed, and of those, only 1 child was documented to have been adherent. This study demonstrates the need for improvement for appropriate pre-travel advice.

It is important to engage the patient in pre-travel counselling as those that do take prophylaxis have a milder course, present with lower parasitemia rates, and if they adhere to the medication as prescribed rarely get malaria [34]. Chemoprophylaxis prescription rates in children are particularly low [26, 27, 35–37]. Some believe parents failing to seek pre-travel advice can be attributed to parents falsely assuming that their child is protected from infection, but in other cases the cost of pre-travel consultations and prophylaxis are barriers [27, 38].

The strengths of our study include the large number of years studied which spans changes in diagnosis techniques and treatment management. Furthermore the population of Toronto is a uniquely international population that represents modern trends in travel, tourism, and immigration. However the data that we include is from one institution and although the largest tertiary paediatric care hospital in the region with experienced infectious disease consultants, we do not present data from other institutions and outpatient clinics that would see returning travelers and vulnerable populations such as immigrants and refugees.

Other implications from this study are that new immigrants/refugees are at high risk for malaria soon after their arrival to Canada. People new to many provinces in Canada, such as Ontario, do not have health care coverage for the first 90 days, in addition there have been heath care cuts to refugee claimant from certain countries [39], which takes away health coverage when these populations are at highest risk of malaria and other tropical diseases. The lack of healthcare coverage may result in delays in diagnosis, more severe disease and undue financial burden.

## Conclusions

Malaria continues to be a significant disease in returning pediatric travelers and immigrant or refugee populations. Continued prevention with appropriate pre-travel advice and increase in physician awareness is required to reduce incidence and decrease delay to treatment.

## Abbreviations

ED: Emergency department
CATMAT: Committee to Advise on Tropical Medicine and Travel
CIHI: Canadian Institute for Health Information
Hgb: Hemoglobin
Hct: Hematocrit
ICD-9/10: International classification of diseases 2009 or 2010
ID: Infectious disease
IQR: Interquartile range
SD: Standard deviation
SickKids: The Hospital for sick children in Toronto, Canada
VFR: Visiting friends or relatives
WHO: World Health Organization.
